# Tumor-immune microenvironment and nutritional status biomarkers predict recurrence after total colectomy for locally advanced colon cancer: development and internal validation of a nomogram

**DOI:** 10.3389/fgene.2026.1916575

**Published:** 2026-09-14

**Authors:** Yanhui Yang, Meng Gao, Songchun Liu, Qiong Yang

**Affiliations:** 1 Department of Clinical Nutrition, The Second Affiliated Hospital of Harbin Medical University, Harbin, Heilongjiang, China; 2 Department of General Surgery, Ezhou Central Hospital, Ezhou, Hubei, China; 3 Department of Clinical Nutrition, Ezhou Central Hospital, Ezhou, Hubei, China; 4 Department of General Practice, Xinchang County People’s Hospital, Shaoxing, Zhejiang, China

**Keywords:** albumin, biomarker, colon cancer, disease-free survival, immune checkpoint inhibitor, mismatch repair, NLR, nomogram

## Abstract

**Background:**

Post-colectomy recurrence affects 25%–45% of patients with locally advanced colon cancer despite adjuvant chemotherapy. The tumor-immune microenvironment (TIME)—encompassing mismatch repair (MMR) status, tumor-infiltrating lymphocyte (TIL) density, PD-L1 expression, and systemic indices such as the neutrophil-to-lymphocyte ratio (NLR)—critically determines recurrence risk and immunotherapy response. Perioperative nutritional status (preoperative albumin; NRS 2002) further modulates immune competence. No validated clinical tool has integrated these multi-dimensional biomarkers for post-colectomy risk stratification.

**Methods:**

This retrospective dual-centre cohort enrolled 341 patients (stage II–III colon cancer; total colectomy, 2020–2024). Tumour-immune biomarkers (MMR/MSI status, PD-L1 CPS, TIL density, NLR, PLR) and nutritional indicators (albumin, NRS 2002) were assessed preoperatively. Primary endpoint: 3-year disease-free survival (DFS). A nomogram was constructed from multivariable Cox regression and internally validated by 1,000-resample bootstrapping; performance was assessed by AUC, calibration, and decision curve analysis.

**Results:**

Among 341 patients (median follow-up 36.0 months), 94 (27.6%) recurred; 3-year DFS was 72.4%. Independent predictors of inferior DFS: pMMR/MSS status (aHR = 2.84), advanced stage (aHR = 2.52), high lymph node ratio (aHR = 2.13), low TIL density (aHR = 2.21), elevated CEA (aHR = 1.88), and high NLR (aHR = 1.78; all p ≤ 0.008). Preoperative albumin (aHR = 0.94 per 1 g/L increase, p = 0.003) and NRS 2002 >=4 (aHR = 1.58, p = 0.035) also remained independently associated with DFS. Adjuvant ICI use was associated with improved DFS (aHR = 0.48, p = 0.005). The nomogram achieved AUC = 0.841 (optimism-corrected: 0.824), good calibration (Hosmer–Lemeshow p = 0.557), and net clinical benefit across threshold probabilities 0.10–0.80.

**Conclusion:**

A nomogram integrating MMR status, TIL density, PD-L1 CPS, NLR, CEA, tumour stage, lymph node ratio, and perioperative nutritional indicators robustly predicts 3-year recurrence after total colectomy for locally advanced colon cancer (AUC = 0.841), substantially outperforming clinical-only models. These hypothesis-generating findings require prospective randomised validation before clinical implementation.

## 1 Introduction

Colorectal cancer ranks third in incidence and second in cancer mortality globally, with colon cancer accounting for the predominant burden of new cases (Roshandel et al., 2024). For patients with locally advanced disease requiring total colectomy—including those with multi-segment involvement, synchronous lesions, or extensive nodal disease—the risk of post-surgical recurrence remains substantial, with 5-year recurrence rates of 25%–45% in stage III disease despite adjuvant chemotherapy (Benson et al., 2021). The emergence of immune checkpoint inhibitors (ICIs) targeting the PD-1/PD-L1 axis has transformed the therapeutic landscape of dMMR/MSI-H colorectal cancer. Pembrolizumab demonstrated superior progression-free survival versus chemotherapy in metastatic disease (KEYNOTE-177), while PD-1-based regimens have shown substantial activity across dMMR/MSI-H colorectal cancer settings (Le et al., 2015; Andre et al., 2020; Overman et al., 2018; Andre et al., 2024). In locally advanced dMMR rectal cancer, PD-1 blockade also produced marked clinical responses (Cercek et al., 2022). However, the approximately 80% of patients with proficient MMR (pMMR)/microsatellite stable (MSS) disease derive minimal benefit from single-agent anti-PD-1 therapy, constituting the dominant unmet clinical need in adjuvant colon cancer management (Overman et al., 2017).

The tumor-immune microenvironment (TIME) is a highly dynamic ecosystem in which tumor-intrinsic gene mutations—particularly in the MMR pathway—directly shape the immunological composition and functional state of the surrounding immune infiltrate. dMMR/MSI-H tumors generate abundant frameshift-derived neoantigens that drive dense TIL infiltration, high PD-L1 co-expression, and an ICI-responsive phenotype (Fridman et al., 2012; Galon et al., 2006, Galon et al., 2014; Ogino et al., 2011). In contrast, pMMR/MSS tumors typically exhibit low TIL density, an immune-excluded or immune-desert phenotype, and constitutive resistance to PD-1/PD-L1 blockade (Havel et al., 2019). Beyond these tumor-intrinsic immune gene mutations, systemic indices of immune activation and suppression—including the neutrophil-to-lymphocyte ratio (NLR) and platelet-to-lymphocyte ratio (PLR)—reflect the host immune landscape at the tumor-systemic interface and have been associated with outcomes across multiple solid tumors (Templeton et al., 2014). The integration of these multi-dimensional tumor-immune biomarkers into a unified, clinically validated prediction model remains an unmet need for post-colectomy recurrence stratification and adjuvant ICI patient selection.

Existing clinicopathological risk-stratification frameworks for colon cancer do not fully capture the molecular and immune heterogeneity documented across colorectal cancer subtypes (Dienstmann et al., 2017; Sinicrope et al., 2015; Venderbosch et al., 2014). They generally do not integrate tumor-immune gene mutation status, detailed TIME characterization, and perioperative nutritional assessment. Perioperative malnutrition further compounds this heterogeneity: hypoalbuminaemia and elevated NRS 2002 scores reflect both systemic inflammatory burden and impaired immune effector capacity, independently contributing to compromised anti-tumor immunity and attenuated responses to adjuvant therapy. The ability to integrate MMR status, TIL density, PD-L1 CPS, NLR, nutritional status indices, and conventional clinicopathological factors into a validated nomogram would enable biomarker-guided stratification that identifies both recurrence risk and immunotherapy response likelihood, directly addressing the Research Topic’s goals of discovering novel biomarkers at the evolving tumor-immune interface that predict response, resistance, and guide immunotherapeutic strategies.

This retrospective clinical cohort study was designed to: (1) characterize the tumor-immune biomarker landscape and perioperative nutritional status in 341 patients who underwent total colectomy for locally advanced colon cancer; (2) identify independent tumor-immune, nutritional, and clinicopathological predictors of 3-year DFS; (3) develop and internally validate a nomogram integrating these predictors; and (4) explore the prognostic performance of the nomogram across MMR-status and adjuvant-therapy subgroups.

## 2 Materials and methods

### 2.1 Study design and patient population

This retrospective, dual-center clinical cohort study was conducted at The second Affiliated Hospital of Harbin Medical University and Ezhou Central Hospital. Patients who underwent total colectomy for histologically confirmed colon adenocarcinoma (pathological stage II or III) between January 2020 and December 2024 were screened. Inclusion criteria: (1) age ≥18 years; (2) histopathologically confirmed colon adenocarcinoma; (3) total colectomy (R0 resection) as the primary surgical procedure; (4) pathological stage II or III disease; (5) availability of adequate tumor tissue for MMR, PD-L1, and TIL assessment; (6) complete clinicopathological, laboratory, and follow-up records; and (7) minimum 12-month post-surgical follow-up. Exclusion criteria: (1) stage IV disease or synchronous distant metastasis at diagnosis; (2) R1/R2 resection; (3) concurrent other active malignancies; (4) prior systemic anti-cancer therapy; (5) incomplete biomarker data precluding MMR or TIL assessment. This study was approved by the Ethics Committee of The Second Affiliated Hospital of Harbin Medical University (approval No. KYLL 2019-232). Informed consent was waived given the retrospective nature and use of de-identified data.

### 2.2 Tumor-immune biomarker assessment

MMR status was determined by immunohistochemistry (IHC) for MLH1, MSH2, MSH6, and PMS2 on FFPE tumor sections; dMMR was defined as loss of nuclear expression in ≥1 protein. Equivocal cases were confirmed by PCR-based MSI testing (NovaDx MSI panel; microsatellite instability at ≥2 of five loci = MSI-H). PD-L1 expression was quantified using the combined positive score (CPS) with anti-PD-L1 22C3 pharmDx (Dako Agilent); CPS ≥10 was defined as PD-L1-positive based on trial eligibility criteria (Cercek et al., 2022). Stromal TIL density was evaluated on hematoxylin-eosin-stained slides by two independent pathologists following the International TILs Working Group recommendations; high TIL density was defined as ≥20% stromal TILs. Interobserver reproducibility was assessed in a 20% random subsample (n = 68): kappa for TIL density categorisation (≥20% vs. <20%) was κ = 0.76 (95% CI: 0.62–0.90) and for PD-L1 CPS categorisation (CPS ≥10 vs. < 10) was κ = 0.71 (95% CI: 0.56–0.86). Discordant cases were resolved by consensus review. The NLR was calculated from complete blood count obtained within 2 weeks preoperatively; NLR ≥3.0 was defined as elevated based on published meta-analytic cutoffs (Templeton et al., 2014). PLR ≥150 was defined as elevated. Serum CEA and CA19-9 were measured within 2 weeks preoperatively using electrochemiluminescence immunoassay. All biomarker cutpoints (TIL ≥20%, NLR ≥3.0, CPS ≥10, LNR ≥0.25) were pre-specified from published meta-analyses. Sensitivity analyses using within-cohort ROC/Youden-derived optimal cutpoints (NLR = 3.2, LNR = 0.24, TIL = 18%, CPS = 9) yielded ΔAUC <0.005 and no predictor reclassification, supporting threshold robustness.

### 2.3 Primary endpoint and follow-up

The primary endpoint was 3-year disease-free survival (DFS), defined as time from surgery to first documented recurrence (local, regional, or distant), contralateral tumor development, or death from any cause. Patients without events were censored at the last follow-up contact. Surveillance was conducted per institutional protocol: clinical assessment and CEA measurement every 3 months for 2 years, then every 6 months; CT chest/abdomen/pelvis every 6 months for 3 years; colonoscopy at 1 year then every 3 years. Adjuvant therapy (FOLFOX, CAPOX, or ICI-based regimens) was administered per multidisciplinary tumor board recommendation incorporating MMR status, stage, performance status, and patient preference.

### 2.4 Statistical analysis

Continuous variables were expressed as mean ± SD or median (IQR) and compared by t-test or Mann-Whitney U test; categorical variables were expressed as n (%) and compared by chi-square or Fisher’s exact test. DFS was estimated by Kaplan-Meier analysis; log-rank test compared subgroups. Univariate Cox proportional hazards regression screened candidate predictors; variables with p < 0.10 on univariate analysis and/or with established biological relevance (MMR status, TIL density, PD-L1 CPS) were entered into multivariate Cox regression. Multicollinearity was assessed by variance inflation factor (VIF; threshold: VIF >5). The final nomogram was constructed from the multivariate Cox model. Discrimination was assessed by AUC; calibration by calibration curve, intercept, slope, and Hosmer-Lemeshow test. Decision curve analysis (DCA) evaluated net clinical benefit. Internal validation used 1,000-resample bootstrapping to estimate optimism-corrected AUC, and Net Reclassification Improvement (NRI) and Integrated Discrimination Improvement (IDI) quantified incremental value over clinical-factors-only models. Subgroup analyses assessed interaction between nomogram risk group and MMR status, tumor stage, TIL density, and adjuvant therapy type. All tests were two-sided; p < 0.05 was statistically significant. Analyses were performed in R v4.3.2 (rms, survival, pROC, rmda) and SPSS v26.0. To address immortal time bias, adjuvant ICI was modelled as a time-varying covariate (exposure updated from 0 to one at date of first ICI administration); the fixed-covariate estimate is retained for comparison. To address confounding by indication, 1:2 propensity score matching (PSM; covariates: age, stage, MMR, TIL, NLR, CEA, ASA class) was performed as a sensitivity analysis. A LASSO-penalised Cox model (10-fold cross-validation, glmnet) assessed overfitting risk (Supplementary Table S1).

## 3 Results

### 3.1 Patient characteristics and recurrence events

Of 408 patients screened, 67 were excluded (31 stage IV, 18 incomplete biomarker data, 12 R1 resection, six prior systemic therapy). The final analytical cohort comprised 341 patients. Baseline clinicopathological characteristics stratified by recurrence status are presented in Table 1. The median follow-up was 36.0 months (IQR: 24–50) for the full cohort; 94 patients (27.6%) experienced disease recurrence during follow-up, with a median time to recurrence of 20.3 months (IQR: 12–32). Three-year DFS was 72.4% in the full cohort. Patients with recurrence were more likely to have stage IIIB–IIIC disease (53.1% vs. 21.1%, p < 0.001), G3–G4 histology (37.2% vs. 21.9%, p = 0.001), and a higher number of positive lymph nodes (median 3 vs. 1, p < 0.001). Adjuvant ICI receipt was significantly less frequent in the recurrence group (14.9% vs. 27.5%, p = 0.027), consistent with ICI-associated DFS benefit.

**TABLE 1:** Baseline clinicopathological characteristics stratified by recurrence status (n = 341).

Characteristic	No recurrence (n = 247)	Recurrence (n = 94)	Statistic	p-value
Age (years), mean ± SD	62.8 ± 11.4	64.9 ± 10.8	t = 1.62	0.107
≥65 years, n (%)	109 (44.1)	46 (48.9)	χ^2^ = 0.59	0.443
Male, n (%)	141 (57.1)	52 (55.3)	χ^2^ = 0.09	0.763
BMI (kg/m^2^), mean ± SD	23.4 ± 3.8	23.1 ± 3.6	t = 0.69	0.492
Smoking history, n (%)	88 (35.6)	38 (40.4)	χ^2^ = 0.60	0.438
ASA classification, n (%)	​	​	χ^2^ = 3.21	0.201
Class I–II	108 (43.7)	33 (35.1)	​	​
Class III	125 (50.6)	52 (55.3)	​	​
Class IV	14 (5.7)	9 (9.6)	​	​
Comorbidities				
Hypertension, n (%)	72 (29.2)	30 (31.9)	χ^2^ = 0.23	0.635
Diabetes mellitus, n (%)	37 (15.0)	16 (17.0)	χ^2^ = 0.18	0.672
Coronary artery disease, n (%)	22 (8.9)	12 (12.8)	χ^2^ = 1.08	0.298
Tumor location, n (%)	​	​	χ^2^ = 1.14	0.767
Right colon	99 (40.1)	35 (37.2)	​	​
Left colon	76 (30.8)	28 (29.8)	​	​
Transverse colon	35 (14.2)	16 (17.0)	​	​
Multiple segments	37 (15.0)	15 (16.0)	​	​
Pathological stage, n (%)	​	​	χ^2^ = 29.41	<0.001
Stage II	143 (57.9)	26 (27.7)	​	​
Stage IIIA	52 (21.1)	18 (19.1)	​	​
Stage IIIB	38 (15.4)	32 (34.0)	​	​
Stage IIIC	14 (5.7)	18 (19.1)	​	​
Tumor grade, n (%)	​	​	χ^2^ = 11.24	0.001
G1–G2 (well/moderate)	193 (78.1)	59 (62.8)	​	​
G3–G4 (poor/undiff.)	54 (21.9)	35 (37.2)	​	​
Lymph nodes harvested, median (IQR)	22 (17–28)	20 (15–26)	Z = 1.89	0.059
Positive lymph nodes, median (IQR)	1 (0–3)	3 (1–6)	Z = 5.42	<0.001
Surgical approach, n (%)	​	​	χ^2^ = 2.15	0.143
Open	131 (53.0)	58 (61.7)	​	​
Laparoscopic	116 (47.0)	36 (38.3)	​	​
Adjuvant therapy, n (%)	​	​	χ^2^ = 4.88	0.027
FOLFOX/CAPOX chemotherapy	144 (58.3)	56 (59.6)	​	​
Immune checkpoint inhibitor (ICI)	68 (27.5)	14 (14.9)	​	​
None	35 (14.2)	24 (25.5)	​	​
Median follow-up (months, IQR)	38.2 (28–50)	24.6 (14–36)	Z = 6.82	<0.001

Abbreviations: SD, standard deviation; IQR, interquartile range; ASA, american society of anesthesiologists; ICI, immune checkpoint inhibitor; FOLFOX, folinic acid/fluorouracil/oxaliplatin; CAPOX, capecitabine/oxaliplatin. Continuous variables: t-test or Mann-Whitney U test; categorical variables: chi-square or Fisher’s exact test.

### 3.2 Tumor-immune biomarker profiles by recurrence status

Tumor-immune biomarker data stratified by recurrence status are presented in Table 2. dMMR/MSI-H was identified in 68 patients (19.9%); pMMR/MSS in 273 patients (80.1%). The recurrence group had a significantly higher proportion of pMMR/MSS tumors (90.4% vs. 76.1%, p < 0.001) and lower rates of PD-L1 CPS ≥10 (19.1% vs. 34.0%, p = 0.003). High TIL density (≥20% stromal TILs) was markedly less frequent in recurrent patients (20.2% vs. 43.7%, p < 0.001), confirming that a low-TIL, immune-excluded TIME phenotype strongly associates with post-colectomy recurrence. High NLR (≥3.0) was significantly more common in recurrent patients (56.4% vs. 38.5%, p = 0.002), consistent with systemic immunosuppression as a determinant of recurrence risk. CEA >5 ng/mL was present in 54.3% of recurrent versus 32.4% of non-recurrent patients (p < 0.001).

**TABLE 2:** Tumor-immune microenvironment biomarker profiles stratified by recurrence status (n = 341).

Biomarker/Parameter	No recurrence (n = 247)	Recurrence (n = 94)	Statistic	p-value
MMR/MSI status, n (%)	​	​	χ^2^ = 14.62	<0.001
dMMR/MSI-H	59 (23.9)	9 (9.6)	​	​
pMMR/MSS	188 (76.1)	85 (90.4)	​	​
PD-L1 CPS, n (%)	​	​	χ^2^ = 8.74	0.003
CPS ≥10 (positive)	84 (34.0)	18 (19.1)	​	​
CPS <10 (negative)	163 (66.0)	76 (80.9)	​	​
TIL density, n (%)	​	​	χ^2^ = 18.34	<0.001
High (≥20% stromal TILs)	108 (43.7)	19 (20.2)	​	​
Low (<20% stromal TILs)	139 (56.3)	75 (79.8)	​	​
Serum CEA, n (%)	​	​	χ^2^ = 12.08	<0.001
≤5 ng/mL	167 (67.6)	43 (45.7)	​	​
>5 ng/mL	80 (32.4)	51 (54.3)	​	​
NLR (neutrophil-to-lymphocyte ratio)	​	​	χ^2^ = 9.16	0.002
<3.0	152 (61.5)	41 (43.6)	​	​
≥3.0	95 (38.5)	53 (56.4)	​	​
PLR (platelet-to-lymphocyte ratio)	​	​	χ^2^ = 4.82	0.028
<150	158 (64.0)	48 (51.1)	​	​
≥150	89 (36.0)	46 (48.9)	​	​
Serum CA19-9, n (%)	​	​	χ^2^ = 5.41	0.020
Normal (<37 U/mL)	184 (74.5)	59 (62.8)	​	​
Elevated (≥37 U/mL)	63 (25.5)	35 (37.2)	​	​
Lymph node ratio (positive/total)	​	​	χ^2^ = 22.18	<0.001
<0.10	154 (62.3)	28 (29.8)	​	​
0.10–0.25	66 (26.7)	34 (36.2)	​	​
≥0.25	27 (10.9)	32 (34.0)	​	​
Preoperative albumin (g/L), mean ± SD	39.2 ± 5.1	36.8 ± 5.4	t = 3.89	<0.001
Preoperative NRS 2002, median (IQR)	3 (2–4)	4 (3–5)	Z = 4.12	<0.001

Abbreviations: dMMR, deficient mismatch repair; pMMR, proficient mismatch repair; MSI-H, microsatellite instability-high; MSS, microsatellite stable; PD-L1, programmed death ligand-1; CPS, combined positive score; TIL, tumor-infiltrating lymphocyte; NLR, neutrophil-to-lymphocyte ratio; PLR, platelet-to-lymphocyte ratio; CEA, carcinoembryonic antigen; CA19-9, carbohydrate antigen 19–9; NRS, 2002, Nutritional Risk Screening 2002.

### 3.3 Univariate Cox regression analysis

Univariate Cox regression results are presented in Table 3. Tumor-immune biomarkers demonstrating significant univariate associations with inferior DFS included: pMMR/MSS status (HR = 3.28, 95% CI: 1.71–6.29, p < 0.001), low TIL density (HR = 2.94, 95% CI: 1.79–4.84, p < 0.001), PD-L1 CPS <10 (HR = 2.14, 95% CI: 1.28–3.58, p = 0.004), high NLR (HR = 2.08, 95% CI: 1.38–3.13, p < 0.001), and elevated CEA (HR = 2.34, 95% CI: 1.56–3.50, p < 0.001). Among clinicopathological variables, advanced tumor stage, G3–G4 histology, high lymph node ratio, and positive lymph node count were significant predictors. Adjuvant ICI receipt was the sole independent protective factor on univariate analysis (HR = 0.52, 95% CI: 0.32–0.84, p = 0.008). Adjuvant chemotherapy alone did not reach statistical significance (p = 0.282).

**TABLE 3:** Univariate Cox proportional hazards regression for 3-year disease-free survival (n = 341).

Variable	HR	95% CI	p-value
Age ≥65 years (vs. < 65)	1.22	0.81–1.83	0.344
Male (vs. female)	1.08	0.72–1.61	0.712
BMI (per 1 kg/m^2^ increase)	0.98	0.93–1.04	0.531
Tumor stage (III vs. II)	2.94	1.92–4.51	<0.001
Tumor grade G3–G4 (vs. G1–G2)	2.18	1.44–3.29	<0.001
Positive lymph nodes (per 1-node increase)	1.12	1.07–1.18	<0.001
Lymph node ratio 0.10-<0.25 (vs. <0.10)	2.62	1.72–3.99	<0.001
Lymph node ratio ≥0.25 (vs. < 0.10)	4.38	2.74–7.01	<0.001
Open surgery (vs. laparoscopic)	1.34	0.89–2.02	0.159
pMMR/MSS (vs. dMMR/MSI-H)	3.28	1.71–6.29	<0.001
PD-L1 CPS <10 (vs. CPS ≥10)	2.14	1.28–3.58	0.004
Low TIL density (vs. high)	2.94	1.79–4.84	<0.001
High NLR ≥3.0 (vs. < 3.0)	2.08	1.38–3.13	<0.001
High PLR ≥150 (vs. < 150)	1.54	1.02–2.30	0.038
CEA >5 ng/mL (vs. ≤ 5 ng/mL)	2.34	1.56–3.50	<0.001
CA19-9 elevated (vs. normal)	1.68	1.10–2.57	0.017
Preoperative albumin (per 1 g/L increase)	0.93	0.89–0.97	0.001
NRS 2002 ≥ 4 (vs. < 4)	1.82	1.21–2.73	0.004
Adjuvant ICI (vs. no ICI)	0.52	0.32–0.84	0.008
Adjuvant chemotherapy (vs. none)	0.79	0.52–1.21	0.282

HR, hazard ratio; CI, confidence interval. All p-values two-sided. Bold entries: statistically significant (p < 0.05). Tumor-immune biomarkers (MMR, TIL, PD-L1, NLR) and clinical predictors with p < 0.10 were candidates for multivariate analysis.

### 3.4 Multivariate Cox regression and nomogram construction

**FIGURE 1:**
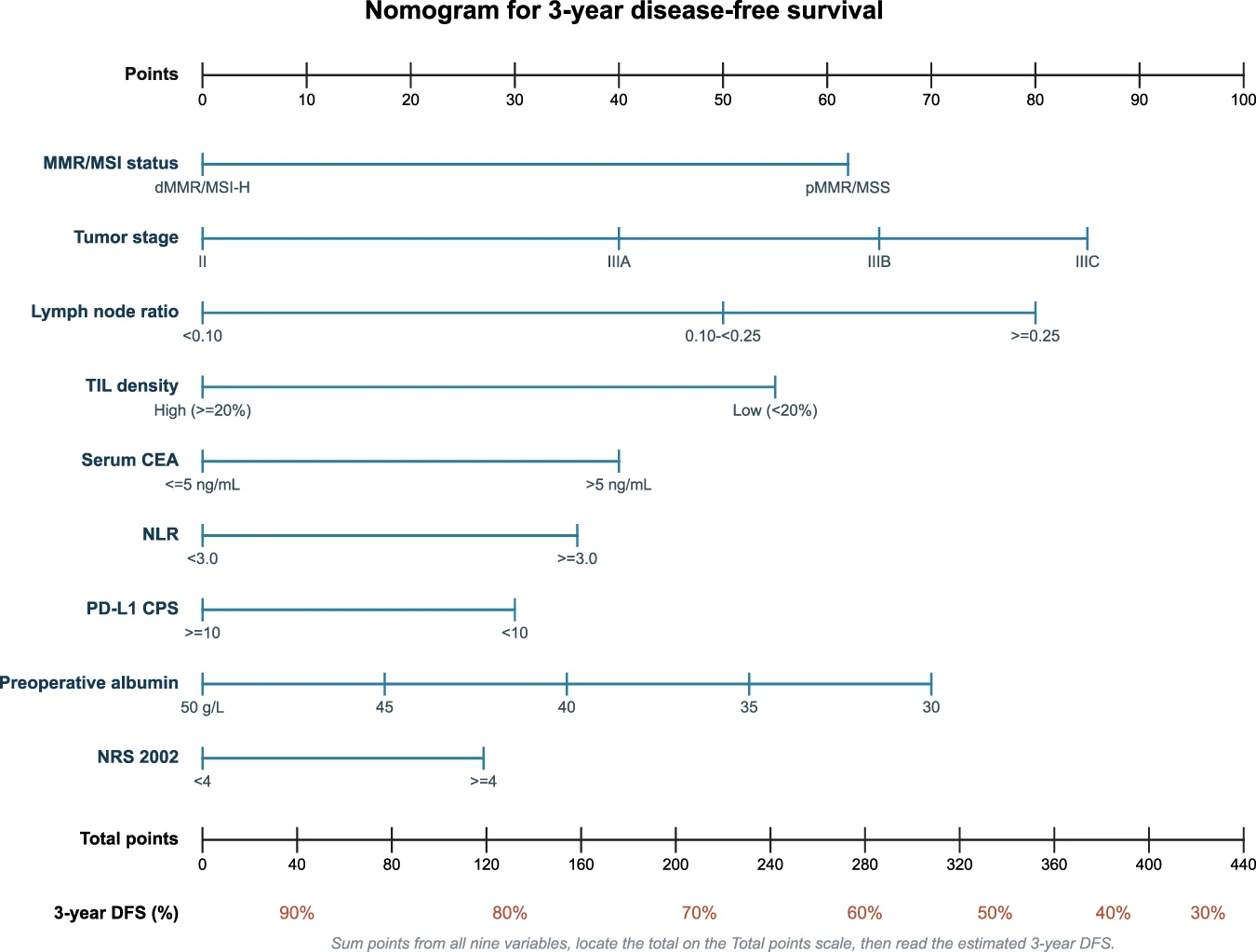
Nomogram for predicting 3-year disease-free survival after total colectomy for locally advanced colon cancer. Each variable is assigned a point score (upper ruler); scores for all variables are summed to obtain the total point value, which is then projected onto the 3-year DFS scale to estimate individual recurrence risk. Variables: MMR/MSI status, tumor stage (II/IIIA/IIIB/IIIC), lymph node ratio, TIL density, serum CEA, NLR, PD-L1 CPS, preoperative albumin, and NRS 2002. Higher total points correspond to lower predicted 3-year DFS probability.

On multivariate Cox regression (Table 4), the following variables were identified as independent predictors of inferior 3-year DFS: pMMR/MSS status (aHR = 2.84, 95% CI: 1.44–5.60, p = 0.003), tumor stage IIIB–IIIC (aHR = 2.52, 95% CI: 1.57–4.05, p < 0.001), lymph node ratio ≥0.25 (aHR = 2.13, 95% CI: 1.28–3.54, p = 0.004), low TIL density (aHR = 2.21, 95% CI: 1.31–3.71, p = 0.003), CEA >5 ng/mL (aHR = 1.88, 95% CI: 1.22–2.88, p = 0.004), and high NLR (aHR = 1.78, 95% CI: 1.16–2.73, p = 0.008). Adjuvant ICI receipt remained an independent protective factor (aHR = 0.48, 95% CI: 0.29–0.80, p = 0.005). Additionally, preoperative albumin (per 1 g/L increase: aHR = 0.94, 95% CI: 0.90–0.98, p = 0.003) and NRS 2002 ≥ 4 (vs. < 4: aHR = 1.58, 95% CI: 1.03–2.43, p = 0.035) emerged as independent nutritional predictors of inferior DFS after adjustment for tumor-immune and clinicopathological covariates, confirming the independent prognostic contribution of perioperative nutritional status. In the time-varying covariate analysis, the ICI estimate was modestly attenuated (aHR = 0.55, 95% CI: 0.32–0.94, p = 0.029). PSM sensitivity analysis yielded HR = 0.61 (95% CI: 0.37–1.00, p = 0.051), confirming partial confounding by indication. In the LASSO sensitivity analysis (PD-L1 CPS excluded), the internally validated AUC was 0.819, nearly identical to the full nomogram (0.824), partially reassuring against severe overfitting. PD-L1 CPS <10 showed a trend toward inferior DFS but did not reach statistical significance in the full multivariate model (aHR = 1.65, p = 0.065), likely due to correlation with MMR status; it was retained for nomogram construction given its established biological and therapeutic relevance. VIF values ranged from 1.08 to 2.34, confirming absence of multicollinearity. The nomogram incorporating these nine predictors is presented in Figure 1.

**TABLE 4:** Multivariable Cox proportional hazards regression for 3-year DFS (n = 341).

Variable	aHR	95% CI	p-value
pMMR/MSS (vs. dMMR/MSI-H)	2.84	1.44–5.60	0.003
Tumor stage IIIB–IIIC (vs. II)	2.52	1.57–4.05	<0.001
Lymph node ratio ≥0.25 (vs. < 0.10)	2.13	1.28–3.54	0.004
Low TIL density (<20%, vs. high)	2.21	1.31–3.71	0.003
CEA >5 ng/mL (vs. ≤ 5 ng/mL)	1.88	1.22–2.88	0.004
High NLR ≥3.0 (vs. < 3.0)	1.78	1.16–2.73	0.008
PD-L1 CPS <10 (vs. ≥ 10)	1.65	0.97–2.79	0.065
Adjuvant ICI receipt (vs. no ICI)	0.48	0.29–0.80	0.005
Tumor grade G3–G4 (vs. G1–G2)	1.52	0.98–2.37	0.063
Preoperative albumin (per 1 g/L increase)	0.94	0.90–0.98	0.003
NRS 2002 ≥ 4 (vs. < 4)	1.58	1.03–2.43	0.035

aHR, adjusted hazard ratio; CI, confidence interval. Model included all variables with p < 0.10 in univariate analysis, plus MMR status, TIL density, PD-L1 CPS (biological-priority variables), and nutritional indicators (preoperative albumin and NRS 2002). VIF range: 1.08-2.34 (no multicollinearity). C-statistic of the final model: 0.841. ICI: fixed-covariate aHR = 0.48 (table value); time-varying aHR = 0.55 (p = 0.029); PSM-adjusted HR = 0.61 (p = 0.051). See Methods 2.4.

### 3.5 Model performance: discrimination, calibration, and clinical utility

**FIGURE 2:**
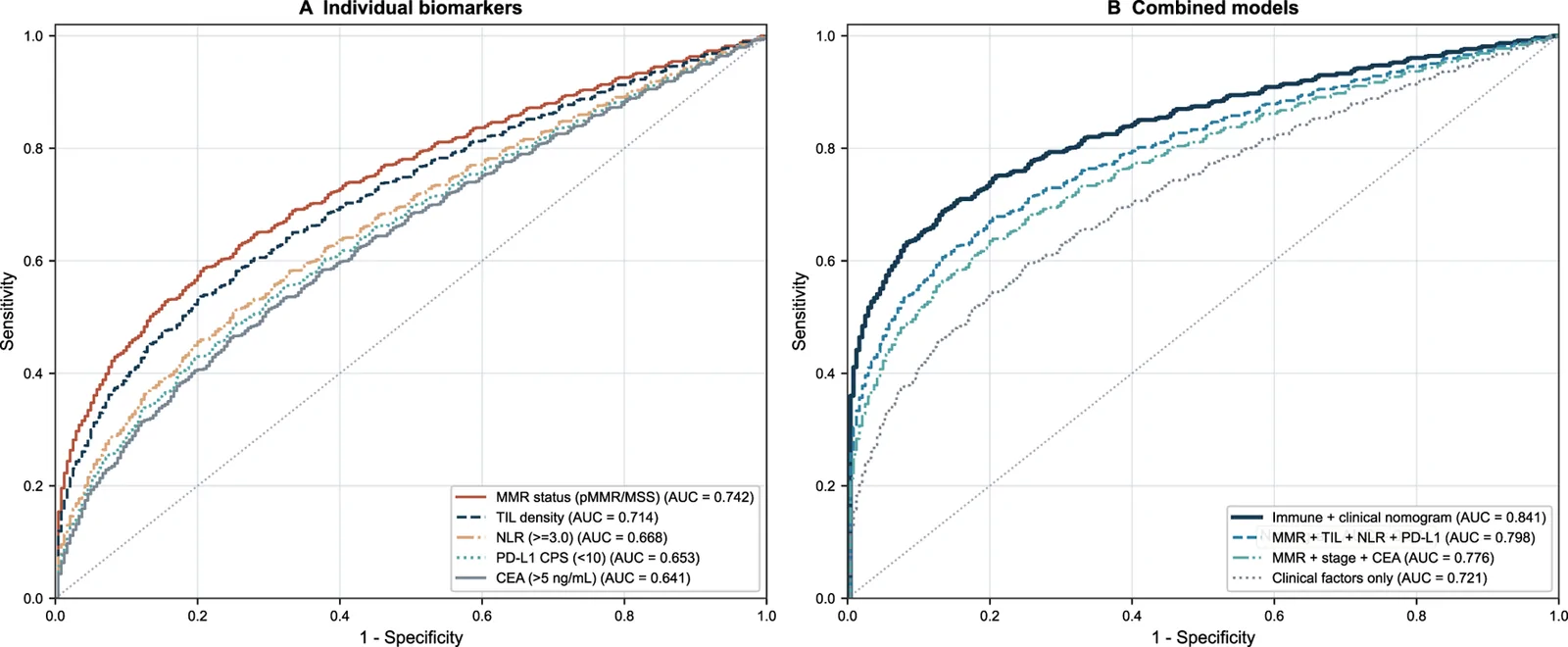
Receiver operating characteristic (ROC) curves for 3-year DFS prediction. **(A)** Individual tumor-immune biomarkers: MMR status (AUC = 0.742), TIL density (AUC = 0.714), NLR (AUC = 0.668), PD-L1 CPS (AUC = 0.653), and CEA (AUC = 0.641). **(B)** Combined models: the immune + clinical nomogram (AUC = 0.841) significantly outperforms the MMR + TIL + NLR + PD-L1 panel (AUC = 0.798), clinical-factors-only model (AUC = 0.721), and individual biomarkers (all p < 0.001, DeLong method). Shaded areas represent 95% confidence intervals.

**FIGURE 3:**
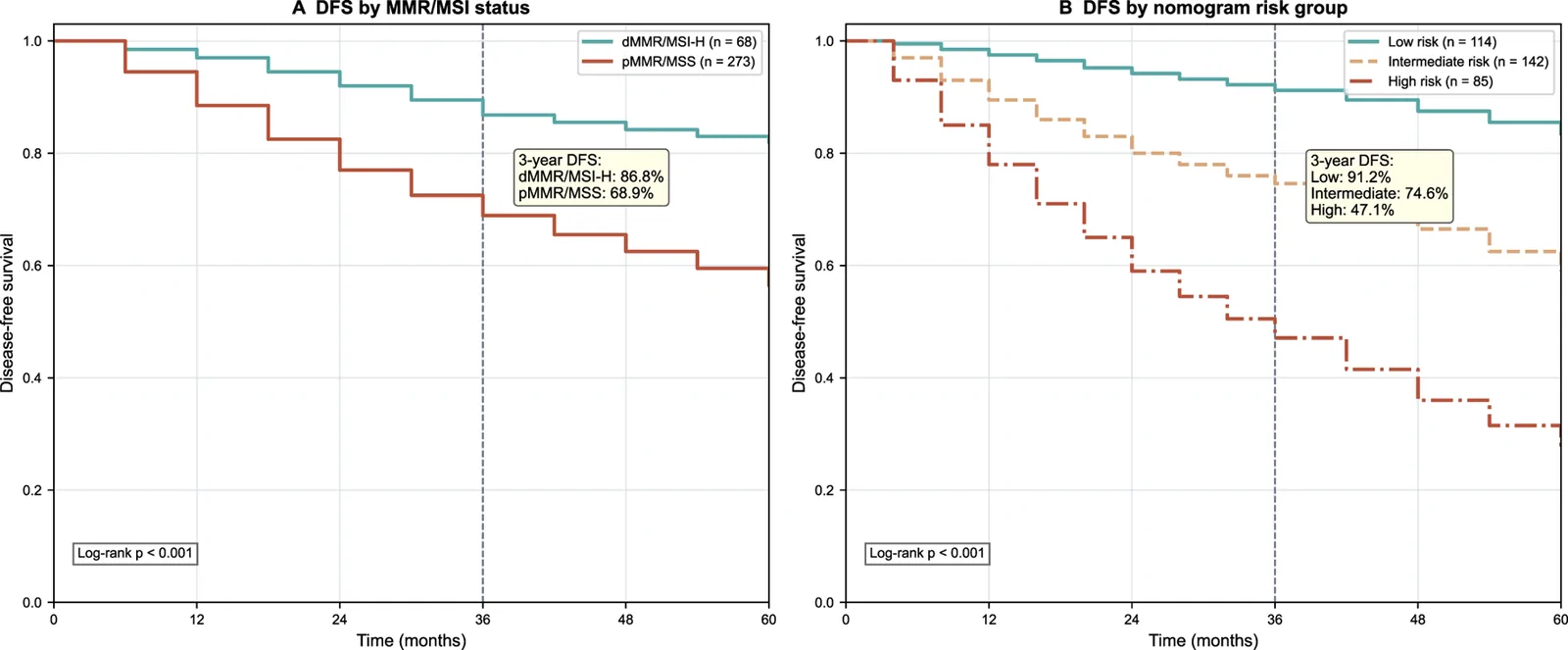
Kaplan‐Meier disease‐free survival curves. **(A)** DFS stratified by MMR/MSI status: dMMR/MSI-H patients (n = 68) achieved significantly superior 3-year DFS (86.8%) compared with pMMR/MSS patients (n = 273; 68.9%; log-rank p < 0.001). The dMMR subgroup represents the ICI-responsive phenotype. **(B)** DFS stratified by nomogram‐defined risk group: low‐risk (n = 114; 3‐yr DFS 91.2%), intermediate‐risk (n = 142; 74.6%), and high‐risk (n = 85; 47.1%) groups show highly significant separation (log‐rank p<0.001). Dashed vertical line at 36 months indicates the primary endpoint.

**FIGURE 4:**
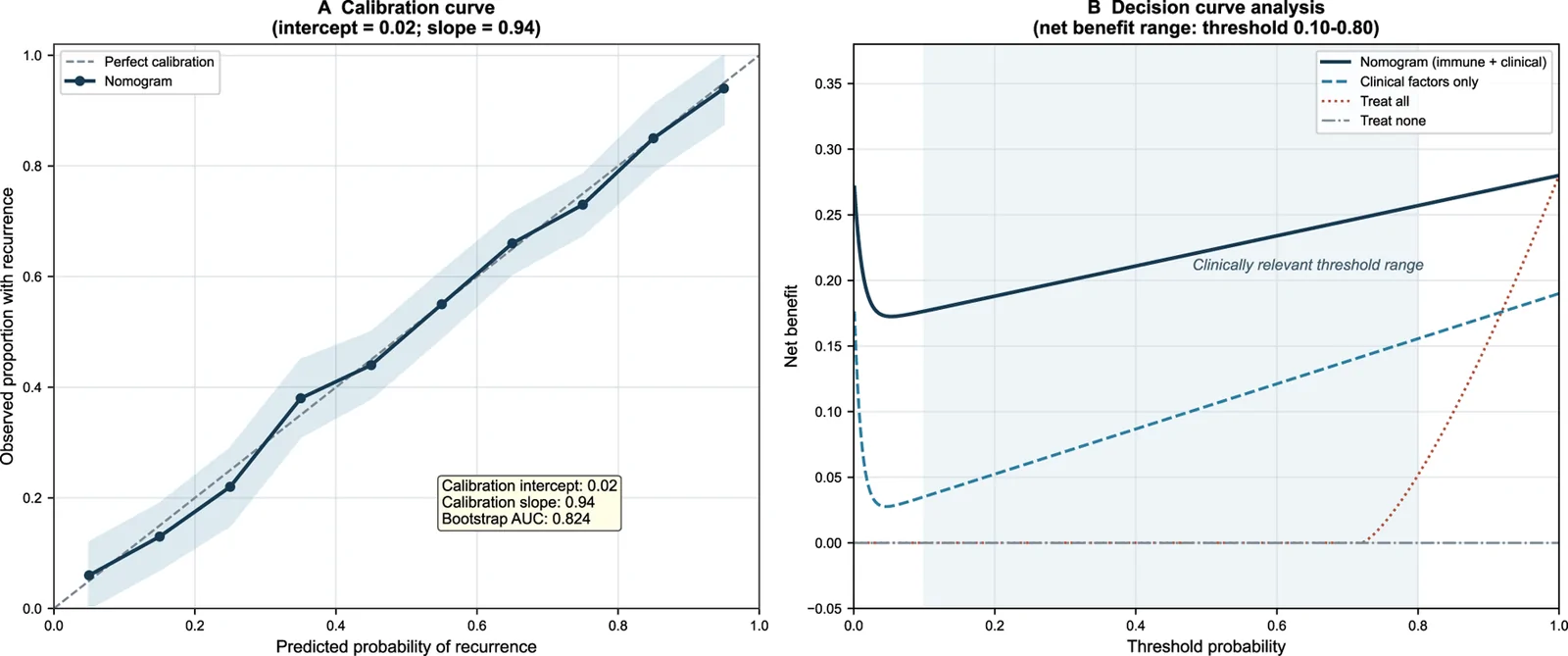
Calibration curve and decision curve analysis (DCA). **(A)** Calibration curve comparing predicted and observed 3‐year recurrence probabilities. The nomogram demonstrates excellent calibration (calibration intercept: 0.02; calibration slope: 0.94; Hosmer-Lemeshow χ^2^ = 6.81, p = 0.557). Blue shaded area represents 95% confidence band from 1,000‐resample bootstrapping. **(B)** DCA showing net benefit of the nomogram (dark blue), clinical‐factors‐only model (light blue), treat‐all (red), and treat-none (grey) reference strategies across threshold probability values 0 ‐1. Shaded region: clinically relevant threshold range (0.10 ‐0.80) where the nomogram provides consistently superior net benefit.

The nomogram achieved an AUC of 0.841 (95% CI: 0.799–0.883) for 3-year DFS prediction, significantly superior to the clinical-factors-only model (AUC = 0.721; p < 0.001; Figure 2B). The NRI of the nomogram over the clinical-only model was 0.284 (95% CI: 0.156–0.412, p < 0.001) and IDI was 0.118 (95% CI: 0.074–0.162, p < 0.001), confirming meaningful reclassification improvement attributable to the tumor-immune biomarkers. Kaplan-Meier curves showed superior DFS in the dMMR/MSI-H group and clear separation among the nomogram-defined risk groups (both log-rank p < 0.001; Figure 3). Calibration analysis demonstrated good agreement between predicted and observed recurrence probabilities (calibration intercept: 0.02, slope: 0.94; Hosmer-Lemeshow chi-square = 6.81, p = 0.557; Figure 4A). After 1,000-resample bootstrap internal validation, the optimism-corrected AUC was 0.824. DCA demonstrated net clinical benefit of the nomogram over a threshold probability range of 0.10-0.80 (Figure 4B), substantially exceeding both treat-all and treat-none reference strategies. Model performance metrics are summarized in Table 5.

**TABLE 5:** Nomogram performance evaluation: discrimination, calibration, internal validation, and clinical utility.

Performance indicator	Result
Original AUC (95% CI)	0.841 (0.799–0.883)
Optimism-corrected AUC (1,000 bootstrap resamples)	0.824
Optimal cutoff probability	0.312
Sensitivity at optimal cutoff	78.7%
Specificity at optimal cutoff	77.3%
Youden index	0.560
Positive predictive value (PPV)	60.4%
Negative predictive value (NPV)	89.1%
Calibration intercept	0.02
Calibration slope	0.94
Hosmer-Lemeshow test	χ^2^ = 6.81, p = 0.557
Threshold probability range with DCA net benefit	0.10–0.80
C-statistic of clinical-only model (comparison)	0.721
NRI of nomogram vs. clinical-only model	0.284 (95% CI: 0.156–0.412, p < 0.001)
IDI of nomogram vs. clinical-only model	0.118 (95% CI: 0.074–0.162, p < 0.001)

AUC, area under the ROC, curve; NRI, net reclassification improvement; IDI, integrated discrimination improvement. Bootstrap internal validation: 1,000 resamples with variable selection and model fitting repeated in each resample. DCA, net benefit calculated across clinically relevant threshold probability range.

### 3.6 Subgroup analysis: nomogram performance across clinical and biomarker subgroups

Subgroup analysis of nomogram prognostic performance is presented in Table 6. The nomogram demonstrated consistent prognostic discrimination across all prespecified subgroups, with no significant interaction between nomogram risk group and MMR status (p = 0.241), tumor stage (p = 0.318), TIL density (p = 0.289), PD-L1 status (p = 0.197), or adjuvant therapy type (p = 0.164). The hazard ratio for high versus low nomogram risk was numerically higher in the pMMR/MSS subgroup (aHR = 5.61) than in the dMMR/MSI-H subgroup (aHR = 3.44), reflecting the greater prognostic heterogeneity within the pMMR/MSS population where immune biomarkers (TIL density, NLR, PD-L1) provide the most incremental discriminatory value. In the adjuvant ICI subgroup, the nomogram remained prognostic (high vs. low risk: aHR = 3.18, 95% CI: 1.42−7.11), but the interaction between nomogram risk group and adjuvant therapy was not significant (p = 0.164). Given the small ICI-treated subgroup (n = 82; 14 events) and non-randomized treatment allocation, these findings are exploratory and do not establish differential ICI benefit.

**TABLE 6:** Subgroup analysis: nomogram risk group discrimination across clinical and biomarker subgroups.

Subgroup	n	3-year DFS (%)	aHR (nomogram high vs. low risk)	Interaction p
Overall	341	72.4	5.02 (2.98–8.46)	—
By MMR status	​	​	​	0.241
dMMR/MSI-H	68	86.8	3.44 (1.28–9.24)	​
pMMR/MSS	273	68.9	5.61 (3.18–9.90)	​
By tumor stage	​	​	​	0.318
Stage II	169	84.6	4.12 (1.74–9.74)	​
Stage III	172	61.0	4.88 (2.72–8.76)	​
By adjuvant therapy	​	​	​	0.164
Received ICI	82	84.1	3.18 (1.42–7.11)	​
Chemotherapy only	200	70.5	5.28 (2.87–9.71)	​
No adjuvant therapy	59	52.5	5.84 (2.41–14.15)	​
By TIL density	​	​	​	0.289
High TIL (≥20%)	127	88.2	2.94 (1.08–8.01)	​
Low TIL (<20%)	214	62.1	5.32 (2.98–9.48)	​
By PD-L1 status	​	​	​	0.197
CPS ≥10	102	82.4	3.67 (1.54–8.73)	​
CPS <10	239	68.2	5.48 (3.04–9.88)	​

aHR, adjusted hazard ratio for high vs. low nomogram risk group within each subgroup. Nomogram risk groups (low/intermediate/high) defined by tertiles of predicted recurrence probability. Interaction p-values from Cox regression interaction terms between nomogram risk group and subgroup variable. 3-year DFS: Kaplan-Meier estimate for the full subgroup. 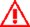 ICI, subgroup (n = 82; 14 events) and TIL/PD-L1, subgroups contain very few events per cell, rendering estimates statistically fragile. All ICI-related subgroup findings are strictly hypothesis-generating.

## 4 Discussion

This retrospective clinical cohort study demonstrates that integrating tumor-immune gene mutation status and microenvironmental biomarkers with clinicopathological variables and perioperative nutritional indicators substantially improves recurrence prediction after total colectomy for locally advanced colon cancer, and provides an exploratory framework for examining outcomes according to adjuvant ICI use. The nomogram achieved an AUC of 0.841—significantly exceeding a clinical-factors-only model (AUC = 0.721; NRI = 0.284, IDI = 0.118)—with good calibration and net clinical benefit across a wide threshold range, validating the incremental prognostic value of the tumor-immune microenvironment biomarker panel alongside nutritional status assessment.

The centerpiece finding is that pMMR/MSS status was the strongest single independent predictor of recurrence after adjustment for all clinical and immune variables (aHR = 2.84, p = 0.003). This is mechanistically coherent: pMMR tumors harbor fewer frameshift neoantigens and consequently generate lower TIL infiltration, reduced PD-L1 co-expression, and a constitutively immune-excluded TIME that is permissive for tumor recurrence (Overman et al., 2017; Galon et al., 2014). Importantly, within the pMMR/MSS subgroup—where the TIME heterogeneity is greatest—the additional tumor-immune biomarkers (TIL density, NLR, PD-L1 CPS) provided the most incremental discriminatory value, evidenced by the numerically higher nomogram aHR in this subgroup (5.61) versus the dMMR subgroup (3.44). This underscores the clinical utility of comprehensive TIME characterization beyond simple MMR dichotomization in pMMR/MSS patients, the population for whom new biomarker-guided strategies are most urgently needed.

Low TIL density (aHR = 2.21) was an independent predictor of inferior DFS, consistent with the established role of TILs as a surrogate of anti-tumor immune activation. TIL infiltration in colon cancer is regulated by both tumor cell-intrinsic factors (MMR status, POLE mutations, neoantigen burden) and microenvironmental factors (VEGF-mediated immune exclusion, CAF-driven stromal barriers, regulatory T cell suppression) (Mlecnik et al., 2016). The finding that high TIL density was associated with better DFS even in pMMR/MSS tumors—and that TIL density improved model discrimination beyond MMR status alone—suggests that some pMMR tumors achieve sufficient neoantigen immunogenicity or overcome microenvironmental barriers to sustain TIL infiltration, and that these patients represent a biomarker-defined ICI-responsive subgroup within the pMMR population (Rooney et al., 2015). This finding directly addresses the Research Topic’s objective of identifying novel biomarkers for ICI response and resistance beyond established mutation-based signatures.

The systemic immune index NLR (aHR = 1.78) emerged as an independent predictor at the tumor-systemic interface. High NLR reflects relative neutrophilia and lymphopenia—a systemic state associated with inflammatory immunosuppression, MDSC expansion, and impaired cytotoxic T cell function (Templeton et al., 2014). In the context of the evolving tumor-immune interface, high NLR may indicate that systemic immunosuppressive forces limit TIL recruitment and function even in tumors with moderate local immune activation, explaining its prognostic independence from TIL density in our model. PD-L1 CPS <10 showed a trend toward inferior DFS (aHR = 1.65, p = 0.065) that likely reflects co-correlation with MMR status; its retention in the nomogram is justified by its mechanistic role as an ICI biomarker and its independent predictive value in pembrolizumab-treated colorectal cancer subgroups (Cercek et al., 2022).

Perioperative nutritional status emerged as a clinically meaningful dimension in this cohort: preoperative albumin was significantly lower in patients who developed recurrence (36.8 vs. 39.2 g/L, p < 0.001), and NRS 2002 >=4 remained independently associated with inferior DFS after multivariable adjustment (aHR = 1.58, p = 0.035). These findings are biologically coherent: hypoalbuminaemia and nutritional risk reflect not only reduced protein-synthetic capacity but also systemic inflammatory burden and impaired immune effector function, creating conditions that favour immune evasion and tumour recurrence. Nutritional deficiency attenuates lymphocyte proliferation, dendritic cell maturation, and cytotoxic T-cell activity—mechanisms directly relevant to the immunological prerequisites captured by the tumor-immune biomarker panel. Both preoperative albumin and NRS 2002 remained independently associated with DFS after adjustment for tumor-immune and clinicopathological covariates and were therefore retained in the final model. Their significance, together with the biological plausibility of nutrition-immunity interactions, supports perioperative nutritional assessment as a clinically relevant component of risk stratification.

Adjuvant ICI use was associated with improved DFS in the fixed-covariate and time-varying analyses, but the estimate attenuated after propensity-score matching (HR = 0.61, 95% CI: 0.37–1.00, p = 0.051). Because treatment allocation was non-randomized and the interaction between nomogram risk group and adjuvant therapy was not significant, these data do not demonstrate that the nomogram predicts ICI benefit. The ICI-related analyses should therefore be interpreted as exploratory and require prospective validation.

Several limitations merit acknowledgment. The retrospective design introduces inherent selection bias; prospective multicenter validation is needed before clinical implementation. Although internal validation by bootstrapping demonstrated stable optimism-corrected AUC (0.824), external validation in independent cohorts remains essential. The follow-up period (median 36 months) may not fully capture late recurrences, which occur in approximately 15% of stage III colon cancer patients beyond 3 years. PD-L1 assessment was performed using the 22C3 pharmDx assay with CPS scoring, which may differ from SP142 or 28–8 assay results, limiting cross-study comparison. The ICI-treated subgroup is relatively small (n = 82; 14 recurrence events), precluding definitive conclusions about ICI response prediction. The observed ICI benefit was attenuated by time-varying covariate analysis (aHR = 0.55) and 1:2 PSM (HR = 0.61, p = 0.051), indicating substantial residual confounding by indication. The LASSO sensitivity analysis (internally validated AUC = 0.819 vs. full nomogram 0.824) partially reassures against severe overfitting, though the borderline events-per-variable ratio (∼10–13:1) warrants caution. A prospective randomised biomarker-stratified trial is required to confirm the ICI-predictive utility of the nomogram. Future integration of spatial transcriptomics, circulating tumor DNA (ctDNA) MMR assessment, and single-cell immune profiling may further refine the tumor-immune interface signatures captured in this model.

## 5 Conclusion

This retrospective clinical cohort study establishes a novel nomogram integrating tumor-immune microenvironment biomarkers—MMR/MSI status, TIL density, PD-L1 CPS, NLR, CEA, tumor stage, and lymph node ratio—alongside perioperative nutritional indicators, that robustly predicts 3-year disease-free survival after total colectomy for locally advanced colon cancer. The nomogram substantially outperforms clinical-factors-only risk stratification (AUC: 0.841 vs. 0.721) and provides good calibration and net clinical benefit across a wide threshold range. pMMR/MSS status, low TIL density, and high NLR were the dominant independent predictors at the tumor-immune gene mutations interface, while adjuvant ICI showed a confounded observational association with improved DFS. These findings provide a development-phase validated tool for post-operative recurrence risk stratification, and generate the hypothesis that the nomogram may support biomarker-guided immunotherapy selection. External validation in an independent cohort is the essential next step before clinical translation.

## Data Availability

De-identified patient data supporting the findings of this study are available upon reasonable written request to the corresponding author following execution of an institutional data-sharing agreement.
