# Correction: Tumor-immune microenvironment and nutritional status biomarkers predict recurrence after total colectomy for locally advanced colon cancer: development and internal validation of a nomogram

**DOI:** 10.3389/fgene.2026.1998945

**Published:** 2026-10-14

**Authors:** Yanhui Yang, Meng Gao, Songchun Liu, Qiong Yang

**Affiliations:** 1 Department of Clinical Nutrition, The Second Affiliated Hospital of Harbin Medical University, Harbin, Heilongjiang, China; 2 Department of General Surgery, Ezhou Central Hospital, Ezhou, Hubei, China; 3 Department of Clinical Nutrition, Ezhou Central Hospital, Ezhou, Hubei, China; 4 Department of General Practice, Xinchang County People’s Hospital, Shaoxing, Zhejiang, China

**Keywords:** albumin, biomarker, colon cancer, disease-free survival, immune checkpoint inhibitor, mismatch repair, NLR, nomogram

In the published article, the **Equal-Contribution** designation for authors Yanhui Yang^†^ and Meng Gao^†^ was inadvertently omitted. The following statement should be added: ^†^These authors contributed equally to this work.

The original version of this article has been updated.

