# Supplementary material for: Tumor-immune microenvironment and nutritional status biomarkers predict recurrence after total colectomy for locally advanced colon cancer: development and internal validation of a nomogram
Source: Front Genet. 2026 Sep 14;17:1916575. doi: 10.3389/fgene.2026.1916575 (PMC13616445; doi:10.3389/fgene.2026.1916575)
Supplement: Supplementary file 1 [file Table1.docx]

**Supplementary Table S1. LASSO sensitivity analysis**

*Tumor-immune microenvironment and nutritional status biomarkers predict recurrence and immunotherapy response after total colectomy for locally advanced colon cancer*

| **Item** | **Reported setting/result** | **Notes** |
| --- | --- | --- |
| Model | LASSO-penalised Cox model | Sensitivity analysis for overfitting risk |
| Cross-validation | 10-fold | Implemented with glmnet |
| PD-L1 CPS | Excluded in LASSO sensitivity analysis | The main nomogram retained PD-L1 CPS for biological/therapeutic relevance |
| Internally validated AUC | 0.819 | LASSO sensitivity analysis |
| Full nomogram optimism-corrected AUC | 0.824 | Reference value reported in the manuscript |
| Interpretation | Nearly identical performance | Supports limited evidence of severe overfitting |

*Note: The author proof reports the LASSO method and summary performance but does not provide individual LASSO coefficients. This supplementary table therefore includes only information explicitly stated in the proof.*
